# Procedural Complications and Inpatient Outcomes of Leadless Pacemaker Implantations in Rural Versus Urban Hospitals in the United States

**DOI:** 10.1002/clc.70081

**Published:** 2025-02-25

**Authors:** Amanda Nguyen, Muhammad Zia Khan, Yasar Sattar, Waleed Alruwaili, Sameh Nassar, Mohamed Alhajji, Bandar Alyami, Joseph Neely, Zain Ul Abideen Asad, Siddharth Agarwal, Sameer Raina, Sudarshan Balla, Bao Nguyen, Dali Fan, Douglas Darden, Muhammad Bilal Munir

**Affiliations:** ^1^ Department of Medicine University of California Davis Medical Center Sacramento California USA; ^2^ Division of Cardiology West Virginia University Heart and Vascular Institute Morgantown West Virginia USA; ^3^ Division of Cardiology University of Oklahoma Oklahoma Oklahoma USA; ^4^ Division of Cardiology Stanford University Stanford California USA; ^5^ Section of Electrophysiology, Division of Cardiology University of California Davis Sacramento California USA; ^6^ Division of Cardiology Kansas City Heart Rhythm Institute Overland Park Kansas USA

**Keywords:** disparities, leadless pacemakers, outcomes, rural, urban

## Abstract

**Background:**

Disparities in invasive cardiovascular care and outcomes in rural and urban hospitals across the United States have been reported. However, studies investigating disparities regarding leadless pacemaker outcomes and complications based on hospital location are lacking.

**Objective:**

To evaluate differences in outcomes and complications related to leadless pacemaker implantations among rural and urban hospitals.

**Methods:**

The National Inpatient Sample was used to identify patients who underwent leadless pacemaker implantations in the United States from 2016 to 2020. Study endpoints assessed included procedural complications and inpatient outcomes of leadless pacemaker implantations among rural and urban hospitals.

**Results:**

From 2016 to 2020, there were a total of 28 340 and 665 leadless pacemaker implantations in urban and rural hospitals, respectively. Baseline characteristics were similar among both groups, with notable exceptions of higher rates of coagulopathies (13.2% vs. 6.8%, *p* < 0.001) and peripheral vascular disorders (10.4% vs. 4.5%, *p* < 0.001) among urban patients. After multivariable adjustment for confounding variables, leadless pacemaker placements occurring in rural hospitals had lower odds of major complications (aOR 0.59, 95% CI 0.41–0.86), but increased odds of inpatient mortality (aOR 1.70, 95% CI 1.21–2.40). Overall, rural leadless pacemaker recipients experienced lower rates of discharge to home, as well as lower costs and length of stay.

**Conclusions:**

A majority of leadless pacemaker implantations occurred in urban hospitals in the United States. Important differences in outcomes were described based on urban and rural hospital location. Further investigation and policy changes are encouraged to promote improved cardiovascular care and outcomes in rural residents.

## Summary

- A majority of leadless pacemaker implantations occurred in an urban hospital setting as compared to a rural hospital.
- Rural leadless pacemaker recipients had similar rates of co‐morbidities, lower rates of major complications, but higher rates of mortality in the crude analysis.
- Rural hospital location was independently associated with increased mortality, decreased length of stay and decreased hospitalization cost after leadless pacemaker implantation.

## Graphical abstract

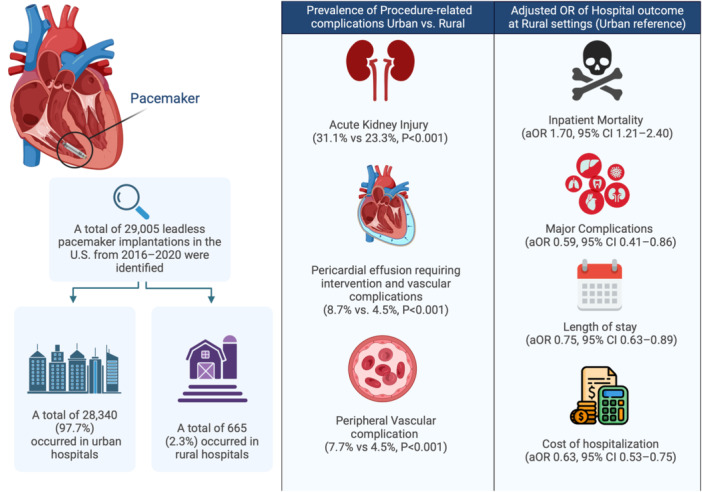

## 1 Introduction

Leadless pacemakers are novel devices that can provide necessary pacing support for select patients who require single chamber ventricular pacemakers. They offer several benefits compared to traditional transvenous pacemakers due to the elimination of complications associated with transvenous leads and pacemaker pockets [1, 2]. Leadless pacemakers may be preferentially and safely placed in target patient populations that have relative contraindications to traditional transvenous pacemakers due to conditions such as venous thromboembolisms, high infection risk, or end‐stage renal disease [3, 4]. On April 6, 2016, the FDA approved the Micra Transcatheter Pacing System, the first FDA‐approved intracardiac leadless pacemaker [5].

Disparities in cardiovascular care and outcomes in rural and urban hospitals across the United States have been reported. Recent studies have demonstrated that rural patients experiencing a variety of cardiac conditions, such as acute myocardial infarction, atrial fibrillation, and valvular diseases are less likely to undergo appropriate cardiac procedures, and are more likely to experience higher inpatient mortality rates than their urban counterparts [6, 7, 8, 9, 10, 11, 12, 13, 14]. It has been postulated that these disparities among rural patients are linked to decreased access to higher level of care, decreased referrals and assessments by cardiac specialists, transportation barriers, etc. [6, 11, 15, 16, 17, 18]. In the realm of cardiac pacing, studies have suggested that most intracardiac and transvenous pacemakers are placed at large, urban teaching hospitals [19, 20]. However, national studies investigating disparities regarding rural versus urban leadless pacemaker outcomes and complications are still lacking.

In this study, we aimed to address this gap in the literature by investigating differences in outcomes and complications related to leadless pacemaker implantations among rural and urban hospital locations in the United States.

## 2 Methods

### 2.1 Data Source

Data from the National Inpatient Sample (NIS) was used for the purpose of our current study. The NIS is a large hospital‐based administrative database that samples inpatient data from 20% of participating hospitals across the nation and is able to estimate > 97% of all U.S. hospitalizations by applying discharge weights. The NIS can be used for computing national estimates of healthcare utilization, costs, trends and outcomes. The database was made possible by a Federal‐State‐Industry partnership sponsored by the Agency for Healthcare Research and Quality (AHRQ) [21].

In our study, we analyzed the NIS database from years 2016 to 2020 for leadless pacemaker device implantations. On April 6, 2016, the FDA approved the first leadless pacemaker device, the Medtronic Micra, which was the only FDA‐approved leadless pacemaker device during the stated study period [5].

The data from the NIS is de‐identified, therefore, the need for informed consent and Institutional Review Board approval is waived [21]. The NIS adheres to the 2013 Declaration of Helsinki for the conduct of human research.

### 2.2 Study Population

Percutaneous leadless pacemaker device implantations were identified using International Classification of Diseases, 10th Revision, Clinical Modification (ICD‐10‐CM) code of 02HK3NZ. Patients younger than 18 years and those with missing demographic data were excluded. The study sample was stratified on the basis of rural versus urban hospital location. As defined by AHRQ, rural and urban hospital location was determined by the Core Based Statistical Area (CBSA), which characterizes micropolitan CBSA and non‐core areas as rural, and metropolitan CBSA as urban [21].

### 2.3 Outcomes

Baseline characteristics, procedural complications, and inpatient outcomes including mortality (reported as a distinct categorical variable in the data set), length of stay, and hospitalization costs were compared in leadless device implantations. We also analyzed major complications (defined as composite of pericardial effusion requiring intervention, and vascular complications, which included arteriovenous fistula, pseudoaneurysm, access site hematoma, retroperitoneal bleeding, and venous thromboembolism), inpatient mortality, prolonged hospital stay (defined as length of stay greater than the median length of stay, > 6 day), and increased hospitalization cost (defined as hospitalization cost > median cost $34 098). NIS provides data on total hospital charges. To estimate hospitalization costs, the cost‐to‐charge ratios based upon CMS reimbursement provided by the Healthcare Cost and Utilization Project were applied to the total hospital charges.

### 2.4 Statistical Analysis

Descriptive statistics are presented as frequencies with percentages for categorical variables and as median with inter‐quartile range (IQR) for continuous variables. Baseline characteristics were compared using a Pearson *X* ^2^ test and Fisher exact test for categorical variables and the Kruskal‐Wallis *H* test for continuous variables. For crude comparison of procedural complications and in‐hospital outcomes among the study groups, the Pearson *X* ^2^ test was used.

To assess the independent association of rural versus urban location with outcomes of mortality, major complications, length of stay and hospitalization costs, a single‐step multivariable logistic regression model was used. Age, sex, race/ethnicity, income, insurance status, and selected Elixhauser comorbidities (Deficiency anemia, Cerebrovascular disorders, Congestive Heart Failure, Chronic pulmonary disease, Coronary Artery Disease, Diabetes, Chronic kidney disease, Hypertension, Liver) were used for adjusted analysis. All these covariates were identified based on prior literature, bivariate analysis and authors best clinical judgment. A *p*‐value of < 0.05 was considered statistically significant. All statistical analyses were performed using SPSS version 26 (IBM Corp, Armonk, NY) and R version 3.6. Because of the complex survey design of the NIS, sample weights, strata, and clusters were applied to raw data to generate national estimates [21].

## 3 Results

A total of 29 005 leadless pacemaker implantations in the United States from 2016 to 2020 were identified in our study after applying the relevant exclusion criteria. Of these procedures, 28 340 (97.7%) implantations occurred in urban hospitals, while 665 (2.3%) occurred in rural hospitals. Baseline characteristics of the study population are shown in Table 1. Women at urban hospitals had significantly increased implantations of leadless pacemakers compared to rural hospitals (44.8% vs. 40.6%, *p* = 0.032). The majority of patients undergoing leadless pacemaker implantation in rural hospitals were white (92.9% vs. 76.1% *p* < 0.001). Overall, the prevalence of co‐morbid conditions was similar between the study groups, with some notable exceptions. Urban patients experienced higher rates of coagulopathies (13.2% vs. 6.8%, *p* < 0.001), peripheral vascular disorders (10.4% vs. 7.5%, *p* < 0.001), and deficiency anemias (5.8% vs. 3.8%, *p* = 0.027). Rural patients experienced higher rates of cerebrovascular disease (13.5% vs. 10.6%, *p* = 0.015). Rural patients also tended to have lower median income, with more patients in the lowest income quartile (53.0% vs. 24.9%, *p* < 0.001), and less patients in the highest income quartile (2.3% vs. 24.3%, *p* < 0.001).

**Table 1:** Baseline characteristics in leadless pacemaker recipients stratified by urban and rural population.

	Urban	Rural	*p* value
Variables no. (%)	28 340 (97.7)	665 (2.3)	
Age (median [IQR]) years	77 (69−85)	78 (70−85)	< 0.001
Females	12 690 (44.8)	270 (40.6)	0.032
Age < 65	4840 (17.1)	105 (15.8)	0.177
65–74	6335 (22.4)	140 (21.1)	
≥ 75	17 165 (60.6)	420 (63.2)	
Race			
White	20 975 (76.1)	590 (92.9)	< 0.001
Black	2770 (10.0)	20 (3.1)	
Hispanic	2085 (7.6)	NR	
Asian or Pacific Islander	885 (3.2)	NR	
Native American	95 (0.3)	15 (2.4)	
Other	760 (2.8)	NR	
**Co‐morbidities**			
Deficiency anemia	1635 (5.8)	25 (3.8)	0.027
Congestive heart Failure	14 805 (52.2)	350 (52.6)	0.842
Connective tissue disorders	835 (2.9)	20 (3.0)	0.927
Chronic pulmonary disease	6915 (24.4)	155 (23.3)	0.517
Cerebrovascular disorders	3000 (10.6)	90 (13.5)	0.015
Coagulopathy	3745 (13.2)	45 (6.8)	< 0.001
Coronary artery disease	12 355 (43.6)	285 (42.9)	0.704
Diabetes mellitus	2760 (9.7)	75 (11.3)	0.186
Hypertension	23 870 (84.2)	575 (86.5)	0.117
Major depression	5650 (19.9)	150 (22.6)	0.215
Hypothyroidism	5650 (19.9)	150 (22.6)	0.095
Liver disease	1775 (6.3)	25 (3.8)	
Obesity	5055 (17.8)	125 (18.8)	0.523
Peripheral vascular disorders	2935 (10.4)	30 (4.5)	< 0.001
Chronic kidney disease	11 580 (40.9)	265 (39.8)	0.6
Pathological weight loss	2975 (10.5)	50 (7.5)	0.013
**Bed size of the hospital**			
Small	2955 (10.4)	NR	< 0.001
Medium	6970 (24.6)	NR	
Large	18 415 (65.0)	650 (97.7)	
**Payee**			
Medicare	23 170 (81.8)	555 (84.7)	< 0.001
Medicaid	1500 (5.3)	10 (1.5)	
Private insurance	2860 (10.1)	75 (11.5)	
Self‐pay	270 (1.0)	NR	
No charge	30 (0.1)	NR	
Other	23 170 (81.8)	555 (84.7)	
**Median income**			
0–25	6965 (24.9)	350 (53.0)	< 0.001
25–50	7305 (26.1)	185 (28.0)	
50–75	6895 (24.7)	110 (16.7)	
75–100	6790 (24.3)	15 (2.3)	

*Note:* For *N* < 11, the absolute numbers are not reported as per Healthcare Cost and Utilization Project recommendations.

Other important procedure‐related complications and inpatient outcomes after leadless pacemaker implantation and stratified on the basis of hospital location are shown in Tables 2 and 3, respectively. The prevalence of major complications, defined as a composite of pericardial effusion requiring intervention and vascular complications, was higher in urban patients compared to their rural counterparts (8.7% vs. 4.5%, *p* < 0.001). Urban patients also experienced higher rates of any peripheral vascular complication (7.7% vs. 4.5%, *p* < 0.001) and acute kidney injury (31.1% vs. 23.3%, *p* < 0.001) compared to rural patients after leadless pacemaker implantation. Inpatient mortality was more prevalent in rural patients undergoing leadless pacemaker implantations compared to urban patients (6.8% vs. 4.9%, *p* = 0.030). Rural patients were also found to have more non‐home discharges (39.5% vs. 33.5%, *p* = 0.002), lower cost of hospitalization ($30 079 vs. $34 217, *p* < 0.001), and shorter length of stay (5 days vs. 6 days, *p* < 0.001).

**Table 2:** Hospital complications in leadless pacemaker recipients stratified by urban and rural population.

	Urban	Rural	*p* value
Variables no. (%)	28 340 (97.7)	665 (2.3)	
**Major complications** a	2460 (8.7)	30 (4.5)	< 0.001
Pericardiocentesis	320 (1.1)	NR	—
Pericardial effusion	990 (3.5)	NR	—
**Any peripheral vascular complication** b	2180 (7.7)	30 (4.5)	< 0.001
AV fistula	70 (0.2)	NR	—
Pseudoaneurysm	240 (0.8)	NR	—
Hematoma	460 (1.6)	NR	—
Retroperitoneal bleeding	100 (0.4)	NR	—
Venous thromboembolism	1405 (5.0)	20 (3.0)	< 0.001
**Acute kidney injury**	8810 (31.1)	155 (23.3)	< 0.001

*Note:* For *N* < 11, the absolute numbers are not reported as per Healthcare Cost and Utilization Project recommendations.

^a^ Defined as a composite of pericardial effusion requiring intervention and vascular complications (AV fistula, pseudoaneurysm, access site hematoma, retroperitoneal bleeding and venous thromboembolism).

^b^ Defined as a composite of AV fistula, pseudoaneurysm, hematoma, retroperitoneal bleeding and venous thromboembolism.

**Table 3:** Hospital outcomes in leadless pacemaker recipients stratified by urban and rural population.

	Urban	Rural	*p* value
Variables no. (%)	28 340 (97.7)	665 (2.3)	
Died at discharge	1395 (4.9)	45 (6.8)	0.030
Discharge disposition			
Home/routine/self‐care	17 930 (66.5)	375 (60.5)	0.002
Nonhome discharges	9015 (33.5)	245 (39.5)	
Resource utilization, median (IQR)			
Cost of hospitalization, $	34 217 (23 381–56 100)	30 079 (22 213−43 827)	< 0.001
Length of stay, days	6 (3−11)	5 (2−8)	< 0.001

**Figure 1:**
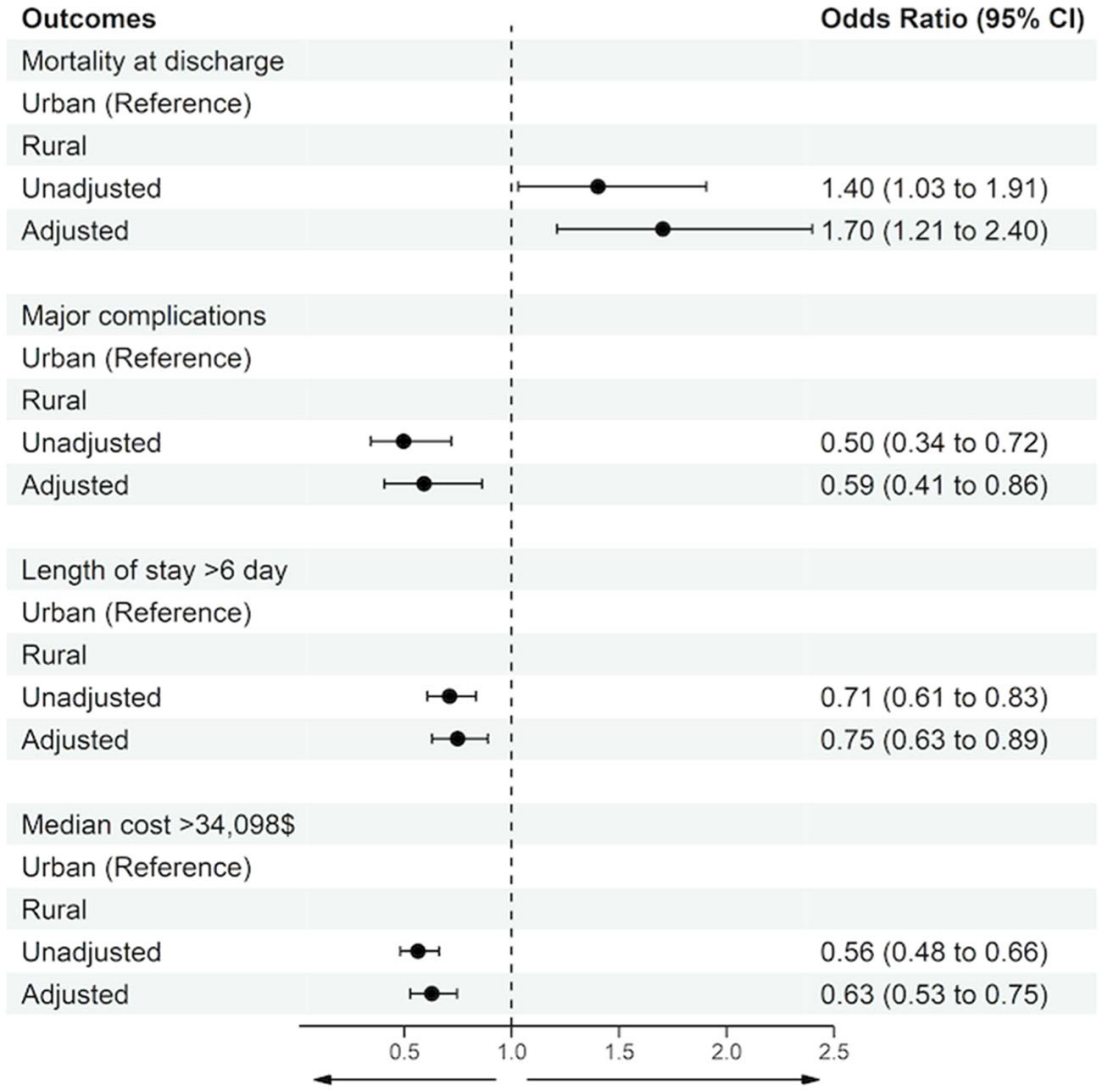
Adjusted association of rural and urban hospital location with outcomes of mortality, major complications, prolonged length of stay, and increased hospitalization costs.

Multivariable models adjusting for potential confounders were constructed to assess the independent association of hospital location with outcomes after leadless pacemaker implantation (Figure 1). After adjustment, rural hospital location for patients undergoing leadless pacemaker implantation was associated with greater inpatient mortality (aOR 1.70, 95% CI 1.21–2.40) but lower rates of major complications (aOR 0.59, 95% CI 0.41–0.86), decreased length of stay (aOR 0.75, 95% CI 0.63–0.89) and decreased cost of hospitalization (aOR 0.63, 95% CI 0.53–0.75).

## 4 Discussion

In this large, contemporary, real‐world cohort of leadless pacemaker implantations in the United States, we report several main findings: (1) a majority of leadless pacemaker implantations occurred in an urban hospital setting as compared to a rural hospital, (2) rural leadless pacemaker recipients had similar rates of co‐morbidities, lower rates of major complications, but higher rates of mortality in the crude analysis, (3) rural hospital location was independently associated with increased mortality, decreased length of stay and decreased hospitalization cost after leadless pacemaker implantation.

Over the past few decades, socioeconomic and racial disparities have been well‐documented in cardiovascular care and outcomes [22, 23, 24, 25]. Health inequities are further apparent based on rural versus urban hospital location and have been specifically demonstrated among more contemporary and advanced cardiac procedures. Loccoh et al. demonstrated decreased procedural rates and increased mortality for rural patients presenting with acute myocardial infarction (AMI), heart failure, or ischemic stroke compared to urban patients [6]. Damluji et al. reported that rural patients in Florida experienced lower transcatheter aortic valve replacement (TAVR) implantations, but higher associated mortality rates when compared to urban patients [14]. Thotamgari et al. reported that rural atrial fibrillation patients had lower left atrial appendage occlusion (LAAO) rates, but significantly higher inpatient mortality rates [26]. Our study is the first nationally representative study to investigate disparities regarding leadless pacemaker outcomes and complications and showed a similar trend of lower implantations and increased mortality in rural hospital recipients as demonstrated by aforementioned studies.

The reported lower leadless pacemaker rates could be related to fewer rural patients being referred to and evaluated by electrophysiologists. This theme has been reported in similar advanced cardiology procedures, such as rural patients being referred less frequently than urban patients for TAVR, implantable cardioverter‐defibrillators (ICD), or cardiac resynchronization therapy [15, 17, 18, 27]. Furthermore, Parkash et al. demonstrated that rural heart failure patients in Canada who were eligible for ICD placement exhibited a higher refusal rate for referral compared to urban patients in the study. Importantly, those rural patients who underwent ICD implantation were found to have significantly reduced mortality rates compared to rural patients who had refused referral [18]. Other healthcare barriers also likely contribute to the health inequities that rural patients experience. Such disparities may include limited healthcare access, poorer health education, lower socioeconomic status, and lower quality of care, expertise or resources associated with rural hospitals [6, 11, 16, 17, 18]. Over the years, the closure of rural hospitals throughout the United States and the challenge of recruiting, training and retaining physicians in rural areas may have also played a part in decreased rates of specialized cardiac procedures, such as leadless device placements [28, 29, 30].

When performed by a specialist with appropriate training, leadless pacemaker implantation is a relatively simple procedure with a swift recovery period. Although leadless pacemakers can be placed in the outpatient setting, patients presenting with acute cardiovascular symptoms or exacerbations may have an urgent inpatient indication for device placement. It is unclear why rural patients in our study experienced shorter length and costs of stay than urban patients. Earlier studies evaluating outcomes after invasive cardiovascular care have shown variability in resource utilization based on urban and rural hospital location. Fogelson et al. reported that patients undergoing TAVR had no significant difference in length of stay between rural and urban hospitals [17]. Thotamgari et al. reported shorter length and costs of stay among rural patients undergoing LAAO [31]. This phenomenon has also been demonstrated in several other national studies in the United States, in which rural patients with AMI experienced slightly decreased or similar length and costs of stay [8, 9, 10, 32]. On the other hand, Moustafa et al. demonstrated that rural patients undergoing catheter ablation for atrial fibrillation, experienced longer length of stay, but lower overall hospitalization costs [26]. More complex cardiac procedures, such as coronary artery bypass graft, have been associated with significantly longer length of stay in rural patients [8].

There may be several reasons behind the lower length of stay reported in our study among rural patients. The increased inpatient mortality rates among rural leadless pacemaker recipients may have contributed to shorter reported duration of stay. Alternatively, these findings may be related to interhospital transfers or selective initial presentation to urban hospitals. As seen in recent years with AMI, patients in rural settings are often transferred out to urban tertiary‐care hospitals that have greater access to invasive cardiac services [6, 33, 34]. It can be speculated that more complicated rural patients requiring higher level of care may have initially presented or been transferred to higher‐volume, more experienced urban hospitals for leadless pacemaker implantation and further delivery of care. The greater rates of major complications, peripheral vascular complications, and acute kidney injury observed in urban patients in our study may be related to a higher baseline acuity of patients presenting to such hospitals. Recent studies have noted increased complication rates in urban patients undergoing cardiac procedures such as catheter ablation, LAAO, and TAVR [17, 26, 31]. Although the feasibility of interhospital transfers has evolved over the years, increasing the advanced cardiac capabilities of rural hospitals associated with a cardiac surgical back‐up may be integral in addressing cardiovascular disparities among their inhabitants. A multifaceted approach to identify and address the variety of healthcare barriers that rural residents face is needed to promote more equitable care.

## 5 Limitations

The results of our current study should be interpreted in the context of the following limitations. First, the NIS relies on administrative coding for disease and procedure identification which may be subject to errors. However, it should be noted that the NIS uses a rigorous data quality control program to minimize miscoding and ensure integrity of data [21]. Second, as the NIS includes data on inpatient stays only, long‐term outcomes and mortality cannot be ascertained. Third, there is no data available on factors that may influence successful leadless pacemaker implantation, such as operator experience or volume of device placement per hospital. Fourth, NIS only reports data on inpatient admissions and does not provide information on outpatient encounters. As leadless pacemaker implantation can be scheduled in the outpatient setting, our findings may not wholly reflect key differences between rural and urban device placement.

## 6 Conclusions

In this large, contemporary, real‐world cohort of leadless pacemaker implantations in the United States, rural patients had lower overall procedural rates compared to their urban counterparts. Rural patients were also noted to have lower length and costs of stay. Despite lower rates of major complications, rural leadless pacemaker recipients had higher rates of mortality. Further investigation is needed to understand the key drivers in outcome disparities among leadless pacemaker patients in rural locations in order to guide policies and programs to mitigate observed inequities.

## Conflicts of Interest

The authors declare no conflicts of interest.

## Supporting information

Supporting information.

## Data Availability

The data that support the findings of this study are available in NIS Database at https://hcup-us.ahrq.gov/db/nation/nis/nisdbdocumentation.jsp. These data were derived from the following resources available in the public domain: HCUP, https://hcup-us.ahrq.gov/db/nation/nis/nisdbdocumentation.jsp.
