# Supplementary figures and images for: Procedural Complications and Inpatient Outcomes of Leadless Pacemaker Implantations in Rural Versus Urban Hospitals in the United States
Source: Clin Cardiol. 2025 Feb 25;48(3):e70081. doi: 10.1002/clc.70081 (PMC11851073; doi:10.1002/clc.70081)

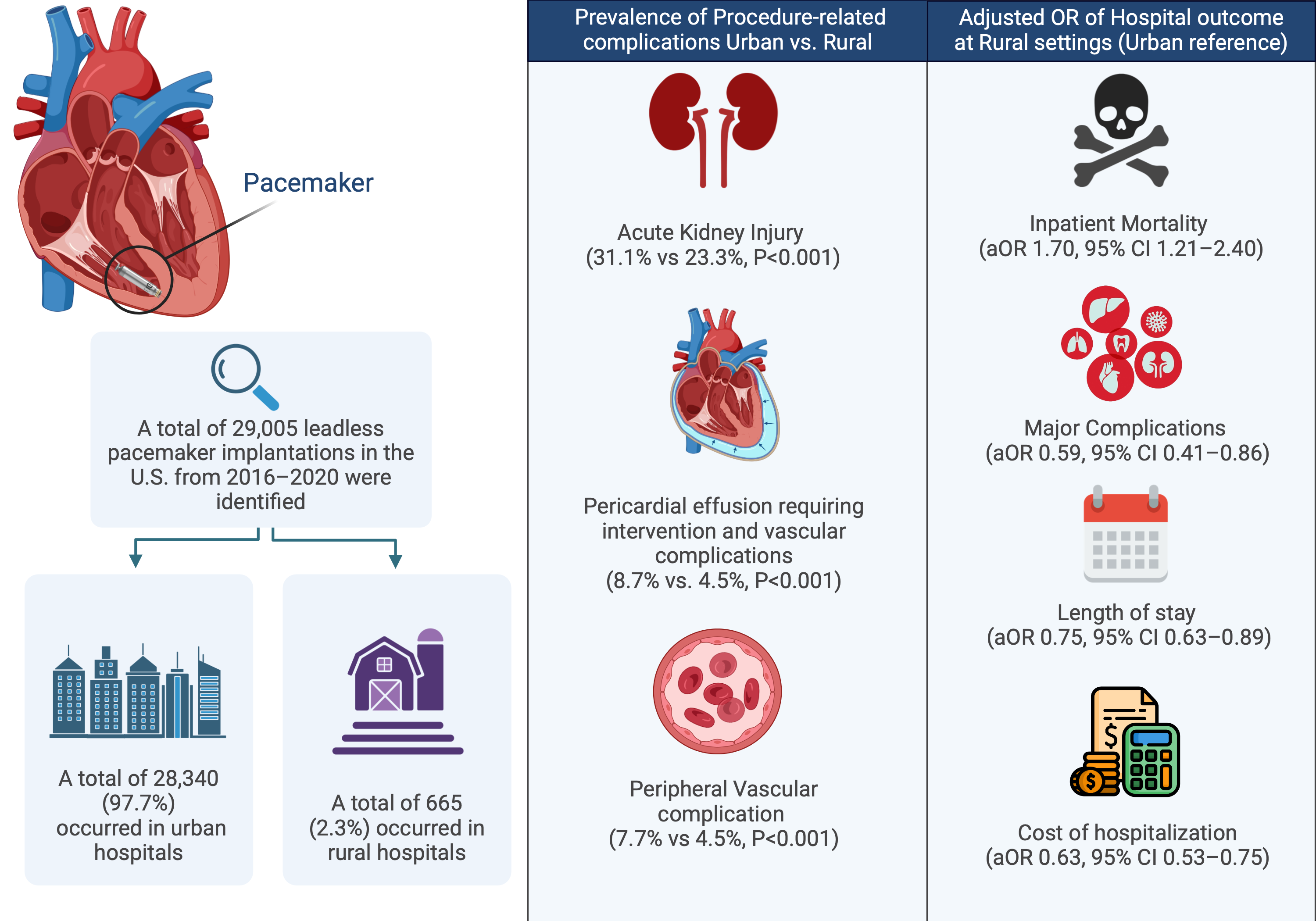

Supplement: Supplementary file 1 — Supporting information. [file CLC-48-e70081-s001.png]
